# The new rehabilitation definition for research purposes could improve rehabilitation description in Cochrane Systematic Reviews

**DOI:** 10.1002/cesm.70000

**Published:** 2024-09-12

**Authors:** Irene Battel, Chiara Arienti, William Levack, Carlotte Kiekens, Stefano Negrini

**Affiliations:** ^1^ Department of Biomedical, Surgical and Dental Sciences University of Milan (La Statale) Milan Italy; ^2^ Department of Biomedical Sciences, Clinical Epidemiology and Research Center Humanitas University Milan Italy; ^3^ Department of Medicine University of Otago Wellington New Zealand; ^4^ Evidence‐Based Rehabilitation Laboratory IRCCS Istituto Ortopedico Galeazzi Milan Italy

**Keywords:** evidence‐based medicine, evidence‐based practice, rehabilitation, research design, systematic reviews as topic

## Abstract

**Introduction:**

In 2022, Cochrane Rehabilitation developed a new definition of rehabilitation for research purposes with 80 global stakeholders, aiming to support and improve the production and reporting of primary and evidence‐synthesis rehabilitation studies.

**Objective:**

1. To compare how Cochrane Systematic Review (CSR) authors describe rehabilitation interventions against criteria derived from the new rehabilitation definition.
2. To assess limitations or gaps in the rehabilitation definition.

**Methods:**

We analysed a sample of 124 randomly selected CSRs tagged in the Cochrane Rehabilitation database. We converted the Cochrane Rehabilitation definition for research purposes into a set of 13 criteria grouped according to the four PICO elements and searched for the corresponding key elements in each CSR. We verified if and where in the review these elements were present. Two reviewers rated each CSR, resolving disagreements with a third author when needed. We analysed the findings using descriptive statistics.

**Results:**

Eight (6.5%) of 124 CSRs met all rehabilitation definition criteria. These were CSRs that investigated the effects of complex rehabilitation interventions. Three (2.4%) CSRs did not meet any PICO elements. Overall, the “Intervention‐General” element and disability criterion had the highest prevalence of absent and unclear reporting, while the “Intervention‐Specific” and “Outcome” elements were most frequently reported, albeit not in the “Description of the intervention” section of the review.

**Discussion:**

This study showed that the key elements of the new rehabilitation definition are almost always reported in publications identified as rehabilitation reviews but not always consistently or clearly. The disability criterion was frequently unreported, given that the main aim of rehabilitation is reducing disability. Also, the main elements of rehabilitation were frequently not reported. We did not find important gaps in the new definition. All elements of the new definition should be considered when writing review protocols and designing strategies and tools on rehabilitation topics.

## 1 INTRODUCTION

In May 2023, the World Health Assembly (WHA) adopted a resolution to strengthen rehabilitation in health systems and to support its wider implementation [1]. This is the consequence of the growing demand for rehabilitation care for all ages in the last decade, due to the demographic trends of an ageing population, an increasing prevalence of noncommunicable diseases, a global escalation of disability and its impact on individuals and societies, and a growing awareness of the beneficial effects of rehabilitation on health and well‐being [2, 3]. Rehabilitation plays a fundamental role in optimizing functioning and supporting active participation in society, with functioning being proposed by the WHA as the third health indicator with mortality and morbidity [4]. The WHA resolution also supports the need for growing research in rehabilitation, and for this reason, there is a need to operationalize what rehabilitation is and is not.

In 2011, the World Health Organization (WHO) defined rehabilitation as “a set of measures that assist individuals who experience, or are likely to experience, disability to achieve and maintain optimal functioning in interaction with their environments” [5]. This definition was changed in 2017 to “a set of interventions designed to optimize functioning and reduce disability in individuals with health conditions in interaction with their environment” [6, 7]. Although these definitions provided a framework for understanding rehabilitative interventions, they did not fit with research needs mostly because they were so broad that very few interventions could be excluded [8]. Since its foundation, Cochrane Rehabilitation faced several challenges due to the lack of a precise rehabilitation definition: creating a database of rehabilitation‐relevant Cochrane Systematic Reviews (CSRs) [9]; collaborating with the WHO to extract the evidence needed to develop the Package of Interventions in Rehabilitation [10]; and in the effort to develop a specific reporting guideline for rehabilitation studies—the Randomised Controlled Trials Rehabilitation Checklist (RCTRACK) [11]. Moreover, it was found that some CSRs have been reported with the term “rehabilitation” in their title even though the included studies were not, from various perspectives, rehabilitative [12]. Researchers, policymakers, and stakeholders rely on databases to learn about current issues in rehabilitation and to access the latest evidence on the effectiveness of rehabilitation interventions. The lack of a comprehensive definition of rehabilitation can hinder the organization of these databases and make it more difficult for people to access the right information in a timely fashion to guide policy development and clinical decision‐making for people with disabilities.

For these reasons, Cochrane Rehabilitation undertook a project to develop a new comprehensive and standardized definition of rehabilitation, aiming to support the implementation of primary studies and synthesis of the current evidence for knowledge translation and rehabilitation research [13, 14]. Unlike prior definitions, this was based on the *Population*, Intervention, Comparison, Outcome (PICO) framework [15] and the International Classification of Functioning, Disability, and Health (ICF) [16]—the World Health Organization classification, which is recognized as the primary reference framework for rehabilitation. The process for establishing the definition comprised four Consensus Meetings and three Delphi rounds with 80 global rehabilitation stakeholders from five continents. As a result of this work, in 2022, rehabilitation was defined as a “Multimodal, person‐centered, collaborative process” (“Intervention‐General”), including interventions targeting a person's “capacity (by addressing body structures, functions, and activities/participation) and/or contextual factors related to performance” (“Intervention‐Specific”), with the goal of “optimising” the “functioning” (“Outcome”) of “persons with health conditions currently experiencing *disability* or likely to experience *disability*, or persons with *disability*” (“Population”). Further details on each element of this definition can be found in the original paper [17] and in Table 1.

**Table 1:** Rehabilitation definition with the description of each term as defined in Negrini et al. [17].

Pico elements	Criteria	Description
Intervention general	Multimodal	Application of more than one intervention or of one intervention with more than one component
	Person‐centered	Interventions are selected and tailored to an individual's needs and engagement, building on and strengthening the resources
	Collaborative	Participation of the person(s) providing the interventions and the person(s) engaged in rehabilitation. The degree of participation and the participants vary according to the health condition(s), the rehabilitation phase (acute, post‐acute, chronic), and the contextual factors, including setting(s) (inpatient, outpatient, home, community). Participation of the person(s) engaged in rehabilitation can be absent at early stages but must gradually develop during the individual continuum of care (rehabilitation process)
	Process	The process includes one or more consecutive rehabilitation cycles (assessment including goal setting, assignment, interventions, evaluation, and repetition if needed) until the optimization of functioning—commonly referred to as the Rehab‐Cycle
Intervention specific	Body structure	Body structures are the anatomical parts of the body, such as organs, limbs, and their components
	Body functions	Body functions are defined as the physiological functions of body systems, including psychological functions
	Activity and participation	Activity is the execution of a task or action by a person. Participation refers to the involvement of a person in everyday situations and in society
	Contextual factors	Contextual factors include personal (that influence how the individual experiences disability) and environmental (the physical, social, and attitudinal environment in which people live and conduct their lives) factors that influence performance (what a person with a health condition does in their usual environment)
Outcome	Optimizing	Improving or maintaining or limiting decline (changing trajectory in terms of deceleration and/or duration) in comparison to the expected (natural) course
	Functioning	Functioning is an umbrella term for body structures and functions, activities, and participation
Population	Health condition	Health conditions include illnesses, injuries, and also physiological changes (e.g., associated with aging or pregnancy) that affect health and functioning
	Experiencing disability	Persons with an impairment(s), activity limitation(s), or participation restriction(s) with potential for resolution of the condition or improvement of functioning
	Likely to experience disability	Probability of disability due to worsening of the health condition or contextual factors, and with a potential for prevention or reduction
	Person with a disability	Persons who have long‐term physical, mental, intellectual, or sensory impairments which in interaction with various barriers may hinder their full and effective participation in society on an equal basis with others (United Nations Convention on the Rights of Persons with Disabilities ‐ UNCRPD), with a potential to avoid or limit decline or optimize functioning

This new definition of rehabilitation requires validation and improvement by comparing it to the existing literature. We decided to perform a first analysis focusing on the CSRs due to their high‐quality standards, and specifically on those already identified as relevant to rehabilitation by Cochrane Rehabilitation [18, 19]

Our aims were:

1. To compare how CSR authors describe rehabilitation interventions against criteria derived from the new rehabilitation definition [17],
2. To assess limitations or gaps in the new rehabilitation definition.

## 2 METHODS

### 2.1 Study design

We performed a methodological study to compare the contents of the rehabilitation‐relevant CSRs against the criteria derived from the new definition published by Cochrane Rehabilitation.

### 2.2 Study selection

CSRs were included in this study if they had been tagged in the Cochrane Rehabilitation database as being about a rehabilitation intervention from the inception of the Cochrane Library to the last update of the database, March 2, 2021 (https://rehabilitation.cochrane.org/evidence). The method for tagging has been previously reported [9, 19] and it consisted of a system that categorizes all Cochrane reviews for: (1) relevance to rehabilitation; (2) relevance to specific professional groups (such as physiotherapists or occupational therapists); (3) broad areas of clinical practice by health condition. The definition we concretely followed during the tagging was “rehabilitation is what rehabilitation professionals do,” even if we recognized that this drives to a circular argument. This was another reason to develop the new definition. Considering the high number of CSRs tagged as being relevant to rehabilitation, we decided to use a sample of CSRs.

The sample size for this study was determined according to the formula of estimation of a proportion 20 samples of 124 CSRs [20]. We used block randomization (10 blocks) to select the CSRs using a specific software (www.random.org).

### 2.3 Data extraction

We analysed Cochrane Rehabilitation's new definition [17] to derive key elements needed to classify a CSR as being a rehabilitation intervention (Table 1). These elements included the four PICO elements of the definition (“Intervention‐General,” “Intervention‐Specific,” “Outcome,” and “Population”), each of which were divided into two to four criteria. In the rehabilitation definition, some elements must be present together (Boolean operator “AND”) and for others one of two or more alternatives are possible (Boolean operator “OR”) (Table 2).

**Table 2:** Description and examples of the rating method used for selecting the Cochrane Systematic Reviews.

Element of definition	Criteria				
Intervention‐General	Multimodal	Person‐centered	Collaborative	Process	Overall score for element
(Boolean operator “AND”)	P	P	P	P	P
	A	P or U	P or U	P or U	A
	P or U	A	P or U	P or U	A
	P or U	P or U	A	P or U	A
	P or U	P or U	P or U	A	A
	U	U	U	U	U
**Intervention‐Specific**	Body structure	Body functions	Activity or participation	Contextual factors	
(Boolean operator “OR”)	P	A or U	A or U	A or U	P
	A or U	P	A or U	A or U	P
	A or U	A or U	P	A or U	P
	A or U	A or U	A or U	P	P
	A	A	A	A	A
	U	U	U	U	U
**Outcome**	Optimizing		Functioning		
(Boolean operator “AND”)	P		P		P
	A		P or U		A
	P or U		A		A
	U		U		U
**Population**	Health Condition	Experiencing disability	Likely to experience disability	Person with a disability	
(Boolean operator “OR”)	P	P	A or U	A or U	P
	P	A or U	P	A or U	P
	P	A or U	A or U	P	P
	P	A	A	A	A
	A	A or U	A or U	A or U	A
	P	U	U	U	U
	U	U	U	U	U

Abbreviations: A, absent; P, present; U, unclear.

Accordingly, we reported absence/presence/unclear for each PICO element as follows:‐ “Intervention‐General” and “Outcome” (Boolean operator “AND”): Scored “present” when all criteria were present, “absent” when at least one criteria was absent, or “unclear” when all the criteria were unclear. (e.g., if a CSR reported on an intervention as being person‐centered, collaborative, and following a process, but did not report the intervention as being multimodal, we scored this as “absent” for “Intervention‐General” as all criteria needed to be met for this element).

Accordingly, we reported absence/presence/unclear for each PICO element as follows:

‐“Intervention‐Specific” (Boolean operator “OR”): Scored as “present” when at least one of the criteria was present, “absent” when all the criteria were absent, or “unclear” when all the criteria were unclear. (e.g., if a CSR reported on an intervention as being designed to alter an aspect of a person's body function, but not their body structure, activity levels, or participation, we scored this as “present” for “Intervention‐Specific” as only one of these criteria had to be met for this element).

‐ “Population” (Boolean operator “OR”): Scored as “present” if the intervention was designed for a specific “Health Condition” and at least one of the three other criteria was present, “absent” when all the criteria were absent, or “unclear” in the other cases (e.g., if a CSR reported on the target population for the intervention as being people with a health condition currently experiencing disability, but not people likely to experience disability or people with a long‐term disability, we scored this as “present” as only one of these three criteria needed to be met).”

We counted a criterion as being met regardless of where the information about that criterion was found in the review. As the Methodological Expectations of Cochrane Intervention Reviews (MECIR) manual [21] and the Cochrane Handbook [22] provide the standards for reporting CSRs, we relied on the statement that “Description of the intervention” in the Background section must accurately describe the intervention being reviewed. Moreover, in scientific writing, the standard structure of the paper allows readers to find information easily in the different manuscript sections, improving accessibility to data. Cochrane reviews are considered to follow the gold standard methodology for systematic reviews. Therefore, we looked at the MECIR manual. We collected data on whether information on each criterion was reported in the “Description of the intervention” in the Background section (“correctly reported”), or in another section (“incorrectly reported”).

Two reviewers (IB and WL) independently rated the selected CSRs and recorded the results on the electronic form from different rehabilitation professions; one reviewer was an experienced rehabilitation researcher (WL), while the other was an early career researcher (IB). Discrepancies were resolved through discussion. If consensus was not reached, a third rater of a third rehabilitation professional with senior clinical and research experience (SN) was involved.

During the analysis of the description of the intervention of the CSRs, the two raters selected also any relevant rehabilitation information that could be categorized under the criteria of the new rehabilitation definition.

### 2.4 Analysis and data interpretation

We reported descriptive statistics on categorical data as frequencies and percentages. We analysed the alignment of the interventions in each review against the criteria derived from the new rehabilitation definition.

We analysed data from the audit process to subjectively evaluate the utility and comprehensiveness of the new definition. We examined instances and patterns of “absent” or “unclear” reporting of criteria associated with the new rehabilitation definition to consider whether reporting in CSRs was sufficiently detailed or whether the new rehabilitation definition needed to be further revised. Likewise, we subjectively evaluated where in the review information about interventions was reported (e.g., “correctly” in the section on “Description of the intervention” or not). We examined differences in scores for each criterion recorded by the two raters to document and analyse possible reasons for disagreements. We also searched the CSRs for any other relevant information used to describe the interventions not considered in the rehabilitation definition.

## 3 RESULTS

We analysed 124 CSRs.

“Intervention‐General” criteria were seldom reported, and rarely reported in the section on “Description of the intervention.” An exception to this was the Process criterion that, when reported (15%; 19/124), was always done correctly in the “Description of intervention” of the CSR (Table 3). “Intervention‐Specific,” “Outcome” and “Population” PICO elements were almost always reported (Table 3). Nevertheless, reporting of information associated with these criteria was frequently not located in the “Definition of the intervention” section of the CSR. For “Intervention‐Specific,” it was often necessary to look at the “Type of outcomes” section in 21% (26/124) of the sample, particularly for reporting on the Body‐structures, Body functions, and Activity/participation criteria. The study of Contextual Factors was occasionally retrieved (4%; 6/124) in the section on “How the intervention might work” (Table 3). In the same section, we also found information reporting on the “Optimizing” (Outcome) and “Functioning” (Outcome) criteria for 71% (89/124) and 55% (68/124) of the samplerespectively. While Health Condition was correctly reported in 98% (100/124) of the CSRs, the information on the Experiencing *disability*, Likely to *experience disability* and *With disability* criteria were most often reported in the “Description of the condition” section (61%, 76/124; 30%, 38/124% and 3%, 4/124, respectively) (Table 3).

**Table 3:** Frequency and Prevalence in reporting the definition criteria in the sample of CSRs.

Macro‐criteria	Criteria	Correct reporting		Incorrect reporting								Absent		Unclear	
		Background		Background						Material and Methods					
		Description of the intervention		Description of the condition		How the intervention might work		Why it is important to do this review		Types of outcome							
Intervention general	Multimodal	9% (11)	5% (6)	‐	‐	4% (6)	0	4% (5)	‐	‐	‐	73% (90)	73% (90)	10% (12)	22% (28)
	Person‐centered	4% (6)		‐		4% (6)		‐		‐		69% (86)		22% (28)	
	Collaborative	10% (13)		‐		15% (19)		6% (8)		‐		52% (64)		16.% (20)	
	Process	15% (19)		‐		‐		‐				65% (81)		19% (24)	
Intervention specific	Body Structure	9% (11)	64% (79)	‐	‐	‐	3% (4)	‐	‐	7% (8)	21% (26)	82% (102)	10% (13)	2% (3)	2% (2)
	Body functions	64% (79)		‐		‐		‐		21% (26)		14% (17)		2% (2)	
	Activity and Participation	7% (8)		‐		‐		‐		2% (3)		85% (106)		6% (7)	
	Contextual Factors	9% (11)		‐		3% (4)		‐		‐		81% (100)		7% (9)	
Outcome	Optimizing	71% (89)	55% (68)	‐	‐	24% (30)	41% (51)	‐	‐	‐	‐	4% (5)	4 (5)%		
	Functioning	55% (68)		‐		41% (51)		‐		‐		4% (5)			
Population	Health Condition	98% (121)	29% (36)	‐	61% (76)	‐	‐	‐	‐	‐	‐	2% (3)	2% (3)		7% (9)
	Currently experiencing disability	25% (32)		61% (76)		‐		‐		‐		5% (7)		7% (9)	
	Likely to experience disability	10% (12)		30% (38)		‐		‐		‐		42% (53)		16% (21)	
	Person with a disability	3% (4)		3% (4)		‐		‐		‐		78% (97)		15% (19)	

*Note*: Correct Reporting when reported in the Description of the intervention of the Background; Incorrect reporting in other section of Background and the Material and Methods; Absent or Unclear reporting.

Overall, we found that eight (6.5%) CSRs met all the PICO elements and criteria of the rehabilitation definition. These eight studies met all criteria of the “Intervention‐General” PICO element and reported a comprehensive rehabilitation approach including multiple interventions. Three (2.4%) CSRs did not meet any PICO elements and criteria. Two of these reviews concerned the use of technology to improve adherence to treatments, but the “Population” element was not described.

With the important exception of the “Outcome” PICO element, the rate of disagreement between the observers was quite high for some criteria (Table 4) and required five meetings to resolve all discrepancies. These disagreements mostly came from a lack of clarity in the report of the CSRs, which resulted in the raters often needing to interpret the intent of the review authors or the context of studies when considering whether or not each criterion was met. For example, we had major disagreements about when a CSR reported on an intervention that targets people with or likely to experience “Disability.” For example, a diagnosis of cerebral stroke or breast cancer could have different sequelae, leading to *disability* or not. It was difficult to determine whether the target “Population” of a CSR was currently experiencing or with *disability* when the authors did not describe the impact of a health condition on functioning and participation.

**Table 4:** Prevalence of disagreements between two raters.

Pico elements	Criteria	Disagreements
Intervention general	Multimodal	9% (11)
	Person‐centered	14% (17)
	Collaborative	10% (12)
	Process	27% (33)
Intervention specific	Body structure	2% (2)
	Body functions	2% (3)
	Activity and participation	11% (14)
	Contextual factors	7% (9)
Outcome	Optimizing	0
	Functioning	0
Population	Health condition	0
	Currently experiencing disability	6% (7)
	Likely to experience disability	10% (13)
	Person with a disability	15% (18)

Most disagreements were in the PICO element “Intervention‐General,” specifically when classifying CSRs against the Process criterion (Tables 4 and 5). The main issue concerned the way the authors described the intervention. Some authors used the words “rehabilitation process” in their CSR but did not provide information to allow us to infer whether they had the same concept of the Process as was described in the rehabilitation definition. Multimodal was another criterion where disagreements commonly occurred. In the “Background” section of CSRs, some authors briefly stated that the Process was part of a complex rehabilitative intervention with interacting factors and interventions, but they did not describe the components of this Process. In the PICO element “Intervention‐Specific,” the disagreements mostly concerned criteria related to activity/participation and contextual factors (Tables 4 and 5). In some CSR, activity/participation were described as outcome measures but in association with quality of life, which was not included as an outcome of interest in the rehabilitation definition. Indeed, most CSRs included quality of life. However, some of the scales ostensibly designed to assess quality of life, included evaluation of components of activity and participation to produce scores, making it difficult to determine how to score against this criterion.

**Table 5:** Summary of the prevalence of the studies categorized according to the groups: (1) Single interventions within the rehabilitation process; (2) Single interventions outside the rehabilitation process; (3) Rehabilitation process, and (4) Uncertain.

		Rehabilitation Process		Single interventions				Uncertain
				Studied within the rehabilitation process		Studied outside the rehabilitation process		
Studies prevalence		10% (13) 95% IC		6% (8) 95% IC		82% (102) 95% IC		1% (1) 95% IC
**Intervention general**	Multimodal	100% (13)	61% (8)	100% (8)	25% (2)	0	0	‐
	Person‐centered	61% (8)		25% (2)		2% (2)		
	Collaborative	61% (8)		37% (3)		28% (29)		
	Process	100% (8)		100% (8)		3% (3)		
**Intervention specific**	Body structure	38% (5)	100% (13)	75% (6)	100% (8)	7% (8)	100% (102)	‐
	Body functions	100% (13)		100% (8)		82% (84)		
	Activity and participation	46% (6)		62% (5)		0		
	Contextual factors	69% (9)		75% (6)		0		
**Outcome**	Optimizing	100% (13)	100% (13)	100% (8)	100% (8)	96% (98)	96% (98)	‐
	Functioning	100% (13)		100% (8)		96% (98)		
**Population**	Health condition	100% (13)	100% (13)	100% (8)	100% (8)	98% (100)	23% (24)	‐
	Currently experiencing disability	76% (10)		62% (5)		20% (21)		
	Likely to experience disability	30% (4)		50% (4)		2% (3)		
	Person with a disability	15% (2)		25% (2)		0		

*Note*: For each category, we report the percentage and frequency of Cochrane Reviews in which the criteria of the new definition [17] were present.

Abbreviation: 95% IC : 95% confidence interval.

## 4 DISCUSSION

According to our aims, this study revealed that overall, the new rehabilitation definition criteria are quite well reported in CSRs, with the important exceptions of the “Intervention‐General” element and the *disability‐related* criteria. The *Intervention‐General* and *disability‐related* criteria are consistently reported by CSRs studying a complex rehabilitation process. We also found that rehabilitation‐relevant information is often sparsely reported in different sections of CSRs, with a relatively low rate of reporting in the expected section of a CSR on the “Description of the intervention,” or not reported in a way that made it easy to evaluate whether key components of rehabilitation were included. These results appear to broadly support the validity of the new rehabilitation definition (most of the elements are usually reported), and we did not find any other elements consistently reported that were missing in the definition. Concurrently, our results suggest that the framework of the new definition of rehabilitation could help to improve reporting in systematic reviews, particularly in CSRs in rehabilitation.

A striking result is that rehabilitation‐related CSRs frequently did not report on *disability*. For example, some CSRs included people with stroke diagnoses, but they did not report the type or severity of the *disability* associated with this health condition. It is recognized that some people with stroke could fully recover and did not necessarily experience *disability* after the event. Reporting on a health condition alone is not sufficient to determine whether a *disability* that requires rehabilitation is present. This is not new, as rehabilitation has always primarily focused on *disability* [22]. There are a couple of possible explanations for this finding: (1) the Cochrane Rehabilitation database includes many CSRs that are not really about rehabilitation and (2) information about rehabilitation interventions is incorrectly reported. The first is possible because the tagging process we followed considered CSRs “of interest for rehabilitation professionals” [19] and the new rehabilitation definition implies that not all of what rehabilitation professionals do is necessarily rehabilitation [17]. Nevertheless, it is possible that the second explanation is more likely, because Cochrane guidelines [21, 22] require review authors to report on *Health Conditions* (almost always present in our results), but not other criteria essential for rehabilitation, such as *disability*. In the future, rehabilitation interventions need to report both the description of the health conditions and the type and degree of *disability*. In addition, these details should not be described only in the population section, but also in the “Background‐Description of Intervention” section of the CSR as they are salient in defining the rehabilitation interventions.

The other important result concerns the most innovative part of the new rehabilitation definition—the overarching concept of rehabilitation (“Intervention‐General”) beyond what has been done (“Intervention‐Specific”) [17]. Only a small proportion of the CSRs sample met all criteria required to fit this aspect of the definition. Of note, these CSRs also described rehabilitation as a complex, multimodal intervention. An intervention is considered complex because of its properties, such as the number of interacting components involved; the range of behavios and processes; the expertise and skills required by those delivering and receiving the intervention, as well as the degree of collaboration and adherence to the intervention; the number of cycles and levels of the process; the different settings; and/or the permitted level of flexibility in the delivery of the intervention or its components [10, 18, 23]. CSRs that focused on complex interventions incorporate all these components, which then met all the criteria contributing to the PICO element “Intervention‐General.”

Few CSRs in our sample explored the effectiveness of interventions that would be considered rehabilitation according to the new definition because of failure to meet the “Intervention‐General” PICO element. There are several potential explanations for this. First, it could be a conceptual issue. If the authors of the CSRs did not conceptualize rehabilitation as a complex intervention, they may not have reported all the components of their interventions. Notably, all CSRs analysed in this study were published before the publication of the new definition of rehabilitation, and no other guidelines for reporting on rehabilitation interventions have previously existed [17]. Second, it is possible that not all CSRs previously tagged by Cochrane Rehabilitation as being rehabilitation reviews meet the criteria for being “rehabilitation” interventions according to the new definition, even if the interventions were correctly reported [17]. The tagging process previously performed by Cochrane Rehabilitation was based on the tautological description of rehabilitation: “rehabilitation interventions are interventions that are provided by rehabilitation professionals” [19, p. 662] and included reviews *of components* of rehabilitation or rehabilitation‐adjacent interventions. Nevertheless, according to the new definition, not all what rehabilitation professionals do is necessarily rehabilitation [17]. Another reason could be a reporting issue. The guidelines for writing a CSR protocol are not specifically designed for rehabilitation interventions and authors should be careful about including all information considered relevant in their field. Hence, the authors may have omitted important information about rehabilitation interventions or could have reported them incorrectly. This reporting issue has been previously proposed in the RCTRACK study, and it is the subject of another Cochrane Rehabilitation project [11, 12, 24].

This study also revealed that the main discrepancy between raters concerned the criteria for “Intervention‐General.” There could be multiple explanations for this finding. One could be the novelty of the rehabilitation definition and some lack of clarity in the definition, complicated by the different backgrounds and experiences of the raters. Moreover, we found that some CSRs briefly gave information about Multimodality, and usually did not provide information on the contributing components, how those interacted with each other, or how they were hypothesized to have an impact on outcomes. Likewise, some authors only named an intervention as the subject of their review without describing its properties, making it difficult to evaluate whether the intervention could be considered a rehabilitation process or not. Indeed, this type of information is very important for determining whether it is to include or group studies in a review or meta‐analysis. Similarly, we previously found a lack of a good description of rehabilitation interventions in RCTs even when these RCTs meet the highest reporting standards for clinical trials [24]. Poor reporting of interventions in RCTs directly impacts the potential for adequate reporting in subsequent CSRs. Cochrane Rehabilitation is currently developing specific reporting guidelines for rehabilitation interventions to overcome this issue [11, 12]. Nevertheless, it is also possible that, in the absence of a definition of rehabilitation sufficient for research purposes, CSR authors work without adequate guidelines.

Most of the CSRs did not report on contextual factors, although these are crucial elements to consider when evaluating disability according to the International Classification of Functioning, Disability, and Health [16, 25]. Moreover, it is well recognized in the literature that environmental and personal factors influence outcomes and must be at least considered and described when they are not the specific focus of the rehabilitation interventions [19, 26, 27]. For this reason, contextual factors are fundamental variables during the rehabilitation intervention and should be described accurately [17].

Conversely, the “Intervention‐Specific” and “Outcome” elements from the new rehabilitation definition were frequently reported on in the CSRs in this study and resulted in few disagreements between raters. These elements of the new rehabilitation definition align with the PICO framework recommended in the Cochrane guideline for writing a protocol [22]. As such, the CSRs authors were likely guided by standard Cochrane methods to include these elements when reporting on their CSR. Unfortunately, they were frequently reported in the wrong place in the paper, confirming on one side their relevance for rehabilitation, and on the other the current misinterpretation of their role in defining the studied intervention as rehabilitation. Overall, these results point to a currently good understanding of many of the details of rehabilitation interventions (“Intervention‐Specific” and “Outcome”), while missing an overarching, global view of rehabilitation (“Intervention‐General”). This again points to the importance of using the new rehabilitation definition to guide reporting in CSRs.

This study also aimed to detect any gaps in the recent rehabilitation definition. We identified that most of the CSRs included “quality of life” (QoL) as an outcome measure, and used validated assessment scales to evaluate this outcome, while the new definition of rehabilitation does not include a focus on QoL as a critical element. The ICF framework used to develop the rehabilitation definition also does not include QoL, and debate on QoL as a measurement construct is ongoing in the world of rehabilitation [28]. In addition, the high rate of discrepancy among raters could be caused by unclear descriptions of some criteria of the new definition. The inexperience of one rater led to misunderstandings of some information in CSRs. Likewise, the different professional backgrounds of raters impacted the assessment of the sample. This supports the importance of a multidisciplinary approach in these evaluations.

There are a few limitations to this study. First, we only analysed CSRs previously identified as being about rehabilitation interventions using different criteria to those of the new rehabilitation definition. This may have meant that we excluded some CSRs from our evaluation that could still have met the criteria of the new rehabilitation definition. To address this issue, we are planning a future study to look at all the other CSRs, particularly those where decisions for inclusion in the Cochrane Rehabilitation database were difficult [19]. Second, some of the authors involved in this analysis were also involved in the development of the new definition of rehabilitation, which may have introduced bias in the development of the database of CSRs from which the study sample was drawn, in the evaluation of the selected CSRs against criteria based on the new definition and in the interpretation of the results. Finally, further analysis of a larger sample of CSRs might alter the frequency of met, unmet, and not reported criteria in this study.

## 5 CONCLUSION

Overall, this study confirmed that the new rehabilitation definition has practical applications—in this case, to help identify and categorize evidence relevant to rehabilitation in a large, general health science database, the Cochrane Library. The new rehabilitation definition should be taken into consideration by researchers so they can avoid omitting key information from their research reports. For example, all systematic reviews on rehabilitation interventions should report on the impact of the target health condition on functioning and disability and should specify the burden of the health condition on activities and social participation. In addition, the new rehabilitation definition can help rehabilitation professionals and researchers retrieve all information required for implementation of an evidence‐based intervention into practice or for replication of previous studies. We did not identify any major gaps in the new rehabilitation definition apart from the possible need to include the concept of quality‐of‐life as a target outcome, which is a subject that requires further studies and debate in the future.

## AUTHOR CONTRIBUTIONS

**Chiara Arienti**: Conceptualization; methodology. **William Levack**: Conceptualization; data curation; formal analysis; methodology; writing—review and editing. **Carlotte Kiekens**: Conceptualization; data curation; writing—review and editing. **Stefano Negrini**: Conceptualization; data curation; formal analysis; methodology; project administration; writing—review and editing.

## PEER REVIEW

The peer review history for this article is available at https://www.webofscience.com/api/gateway/wos/peer-review/10.1002/cesm.70000.

## Data Availability

The data that support the findings of this study are available from the corresponding author upon reasonable request.
